# Supplementary material for: Resolving the full spectrum of human genome variation using Linked-Reads
Source: Genome Res. 2019 Apr;29(4):635–45. doi: 10.1101/gr.234443.118 (PMC6442396; doi:10.1101/gr.234443.118)
Supplement: Supplemental Material [file supp_gr.234443.118_Supplemental_longranger-2.2.zip › longranger-2.2.tar.bz2!/lr2.2-public/lib/stan/cmdstan/stan/doc/api/html/classstan_1_1io_1_1csv__writer.html]

Stan: stan::io::csv\_writer Class Reference


|  |  |
| --- | --- |
|  | Stan  2.5.0  probability, sampling & optimization |


- Main Page
- Related Pages
- Namespaces
- Classes
- Files

- Class List
- Class Index
- Class Hierarchy
- Class Members

All Classes Namespaces Files Functions Variables Typedefs Enumerations Enumerator Friends Macros Pages

- stan
- io
- csv\_writer

Public Member Functions |
List of all members

stan::io::csv\_writer Class Reference

Writes Stan variables in comma-separated-value format to an output stream.
More...

`#include <csv_writer.hpp>`

|  |  |
| --- | --- |
| Public Member Functions | |
| void | comma () |
|  | Write a comma. More... |
|  | |
|  | csv\_writer (std::ostream &o) |
|  | Construct a CSV writer that writes to the specified output stream. More... |
|  | |
| void | newline () |
|  | Write a newline character. More... |
|  | |
| void | write (int n) |
|  | Write a value. More... |
|  | |
| void | write (double x) |
|  | Write a value. More... |
|  | |
| template<int R, int C> | |
| void | write\_row\_major (const Eigen::Matrix< double, R, C > &m) |
|  | Write a value. More... |
|  | |
| template<int R, int C> | |
| void | write\_col\_major (const Eigen::Matrix< double, R, C > &m) |
|  | Write a value in column-major order. More... |
|  | |
| template<int R, int C> | |
| void | write (const Eigen::Matrix< double, R, C > &m) |
|  | |
| void | write (const std::string &s) |
|  | Write a value. More... |
|  | |

## Detailed Description

Writes Stan variables in comma-separated-value format to an output stream.

All output is written to the same line and separated by commas. Use the `newline()` method to advance to the next line of CSV output.

Definition at line 23 of file csv\_writer.hpp.

## Constructor & Destructor Documentation

|  |  |  |  |  |  |
| --- | --- | --- | --- | --- | --- |
| stan::io::csv\_writer::csv\_writer | ( | std::ostream & | *o* | ) |  |

inline

Construct a CSV writer that writes to the specified output stream.

Parameters
:   |  |  |
    | --- | --- |
    | o | Output stream on which to write. |

Definition at line 51 of file csv\_writer.hpp.

## Member Function Documentation

|  |  |  |  |  |
| --- | --- | --- | --- | --- |
| void stan::io::csv\_writer::comma | ( |  | ) |  |

inline

Write a comma.

On the first call do nothing, subsequently write a comma.

Definition at line 36 of file csv\_writer.hpp.

|  |  |  |  |  |
| --- | --- | --- | --- | --- |
| void stan::io::csv\_writer::newline | ( |  | ) |  |

inline

Write a newline character.

End a line of output and start a new line. This method needs to be called rather than writing a newline to the output stream because it resets the comma flag.

Definition at line 63 of file csv\_writer.hpp.

|  |  |  |  |  |  |
| --- | --- | --- | --- | --- | --- |
| void stan::io::csv\_writer::write | ( | int | *n* | ) |  |

inline

Write a value.

Write the specified integer to the output stream on the current line.

Parameters
:   |  |  |
    | --- | --- |
    | n | Integer to write. |

Definition at line 76 of file csv\_writer.hpp.

|  |  |  |  |  |  |
| --- | --- | --- | --- | --- | --- |
| void stan::io::csv\_writer::write | ( | double | *x* | ) |  |

inline

Write a value.

Write the specified double to the output stream on the current line.

Parameters
:   |  |  |
    | --- | --- |
    | x | Double to write. |

Definition at line 89 of file csv\_writer.hpp.

template<int R, int C>

|  |  |  |  |  |  |
| --- | --- | --- | --- | --- | --- |
| void stan::io::csv\_writer::write | ( | const Eigen::Matrix< double, R, C > & | *m* | ) |  |

inline

Definition at line 133 of file csv\_writer.hpp.

|  |  |  |  |  |  |
| --- | --- | --- | --- | --- | --- |
| void stan::io::csv\_writer::write | ( | const std::string & | *s* | ) |  |

inline

Write a value.

Write the specified string to the output stream with quotes around it and quotes inside escaped to double quotes.

Parameters
:   |  |  |
    | --- | --- |
    | s | String to write. |

Definition at line 146 of file csv\_writer.hpp.

template<int R, int C>

|  |  |  |  |  |  |
| --- | --- | --- | --- | --- | --- |
| void stan::io::csv\_writer::write\_col\_major | ( | const Eigen::Matrix< double, R, C > & | *m* | ) |  |

inline

Write a value in column-major order.

Write the specified matrix to the output stream in column-major order.

Parameters
:   |  |  |
    | --- | --- |
    | m | Matrix of doubles to write. |

Definition at line 122 of file csv\_writer.hpp.

template<int R, int C>

|  |  |  |  |  |  |
| --- | --- | --- | --- | --- | --- |
| void stan::io::csv\_writer::write\_row\_major | ( | const Eigen::Matrix< double, R, C > & | *m* | ) |  |

inline

Write a value.

Write the specified matrix to the output stream in row-major order.

Parameters
:   |  |  |
    | --- | --- |
    | m | Matrix to write. |

Definition at line 104 of file csv\_writer.hpp.

---

The documentation for this class was generated from the following file:

- src/stan/io/csv\_writer.hpp


---

[ Stan Home Page ]

*© 2011–2014,
Stan Development Team.*
